# The role of heterocyclic aromatic amines in colorectal cancer: the evidence from epidemiologic studies

**DOI:** 10.1186/s41021-021-00197-z

**Published:** 2021-06-07

**Authors:** Loïc Le Marchand

**Affiliations:** Epidemiology Program, University of Hawaii Cancer Center, 701 Ilalo Street, Honolulu, HI 9681 USA

**Keywords:** Colorectal Cancer risk, Heterocyclic amines, Dietary carcinogens, Epidemiology, Genetic susceptibility, Well-done meat

## Abstract

Since Dr. Sugimura’s discovery of heterocyclic aromatic amines (HAA) in broiled fish, many epidemiological studies have been conducted to investigate their role in human cancers, often focusing on colorectal cancer. The difficulty in measuring HAA exposure from meat and fish intake in these studies has resulted in inconsistent findings. Because studying individuals who may be particularly susceptible to the carcinogenic effects of HAA might facilitate the demonstration of a link with cancer, multiple studies have focused on individuals with the high activity phenotype for CYP1A2 and/or NAT2, the two main metabolic enzymes involved in the bioactivation of HAA. These investigations have also yielded inconsistent results. Two recent large pooled analyses of colorectal cancer studies have helped clarify the overall evidence. One was conducted in whites and reported no interaction of red meat intake and NAT2 genotype on risk in Whites. The other was conducted in Japanese and African Americans, two populations with high rates of the disease and a prevalence of the at-risk rapid NAT2 phenotype 10- and 2-fold greater than in whites, respectively. In those groups, a significant interaction was found, with the association of red meat with colorectal cancer being strongest among individuals with the rapid NAT2 phenotype, intermediate among those with the intermediate phenotype and not significant among those with the slow NAT2 phenotype. Recent research on biomarkers has focused on PhIP hair content, as a marker of exposure to HAA, and on DNA adducts using new sensitive quantitative methods, as markers of early biological effects. These advances, when brought to bear, may contribute greatly to the further elucidation of the carcinogenicity of HAA in humans.

## Discovery of HAAs and their carcinogenicity

Making an analogy between cigarette smoke and the enticing smell emanating from his wife’s kitchen, Prof. Takashi Sugimura astutely wondered one day whether smoke from broiled fish also contained mutagens [1]. Confirming his hunch, he demonstrated for the first time that smoke from broiled fish showed strong mutagenicity in *Salmonella typhimurium* TA98, launching a field of study that spanned over four decades [1]. Several new mutagens belonging to the HAA class of chemicals were then identified from the pyrolysis of amino acids and proteins at the surface of meat and fish cooked at high temperature [1]. The animal studies that followed showed that these compounds are carcinogenic in rodents and monkeys [1]. To act as complete carcinogens, HAAs were shown to require metabolic activation by cytochrome P450 (CYP) 1A2 (and to a lesser degree by CYP1A1, B1 and 3A4) in the liver, followed by a second metabolic step performed by *N*-acetyl-transferase (NAT), which is mainly expressed in the liver and intestinal epithelium [2]. I review here the epidemiological evidence for a role of HAA in the causation of human cancer and, because colorectal cancer (CRC) is the organ site that is the most relevant and has received the most attention, I focus on this cancer.

### Difficulty in measuring HAAs in diet

Many epidemiological studies have investigated the association reported for meat consumption and risk of several cancers of the colorectum, breast and prostate [2]. A 2015 IARC expert report concluded that consumption of processed meat was “carcinogenic to humans” and that of red meat “probably carcinogenic to humans”; consumption of both meat types was associated with an increased risk of CRC [3]. Although several classes of carcinogens and multiple mechanisms are likely to be at play, much attention has focused on the role of HAA in cancer development through eating meat cooked well-done [2, 3]. The majority of studies that investigated consumption of well-done meat or meat prepared by high-temperature methods (barbecuing, pan-frying, broiling or grilling) reported a positive association; however, some studies found no association (reviewed in references 2 and 3). Fewer investigators have attempted to quantify intake of specific HAAs in the diet of their participants. This turned out to be particularly challenging due to the great variation that exists in HAA formation on the surface of meat and fish during cooking. The extent of exposure to humans depends on: the type of meat and fish consumed; mode, temperature and duration of cooking; use of gravy, marinade or sauce; and whether pan residue or the skin is ingested [2]. These parameters can lead to differences in HAA concentrations in the diet by more than a 100-fold. Some of the detailed dietary studies of CRC or its precursor, adenoma, have reported an association [4–7], but some have not [8, 9]. The uncertainties in HAA concentrations in the diet likely result in a poor assessment of usual exposure to these compounds and probably explain the inconsistency in the epidemiological data. Thus, overall, the dietary studies have been suggestive but inconsistent.

### Studies of genetically susceptible subgroups and populations

Given the difficulties in assessing dietary exposure to HAAs through dietary questionnaires, it was proposed that focusing on individuals who can be a priori inferred to have a heightened susceptibility to the carcinogenic effect of HAAs might facilitate the demonstration of a link with human cancer. As mentioned above, CYP1A2 and NAT2 has been shown to play an important role in the bioactivation of HAAs [2]. The activity of each enzyme shows a high inter-individual variation and can be measured by dosing individuals with caffeine and measuring urinary metabolites. These two enzymes are modulated by genetic polymorphisms and, in the case of CYP1A2 is inducible by lifestyle factors (e.g., smoking). Thus, both genetics and lifestyle may contribute to inter-individual differences in susceptibility to HAAs. Two case-control studies have suggested that the combination of high CYP1A2 and high NAT2 activity is a risk factor for CRC or adenoma in individuals exposed to HAAs through the regular consumption of well-done meat [10–12]. In one study, the observed association was limited to smokers which is biologically plausible, since smoking induces CYP1A2 [11, 12]. A third study failed to show a modifying effect of NAT2 or CYP1A2, also measured by caffeine phenotyping, or an association with HAA intake with risk of adenoma [13].

A larger number of studies have focused on NAT2, red meat intake and CRC. The slow activity phenotype for this enzyme varies widely across populations, from ~ 5% in Eskimos to 10% in Japanese, 50% in Europeans and 90% in North Africans [14] Interestingly, the populations with the highest frequencies for the rapid acetylator phenotype also have the highest CRC incidence rates reported in the world (Alaskan Natives and Japanese), and those with the lowest rapid acetylator phenotype frequencies have low CRC rates (in North Africa) [15, 16]. Moreover, the temporal trends of increasing CRC rates in Japan have closely paralleled the increase in red meat intake with a 20-year lag [17]. That NAT2 phenotype may modulate the association of red meat intake with CRC was suggested by an ecologic study that showed that the correlation that exists between country-specific per capita meat consumption and CRC incidence is significantly increased when considering the population-specific prevalence of the *N*-acetylation phenotype [18].

More than 25 single-nucleotide polymorphisms that modify NAT2’s catalytic activity for HAAs have been identified in the *NAT2* gene. Several combinations of SNPs have been proposed to classify *NAT2* genotypes and best infer the phenotype. A panel of seven SNPs for *NAT2* has been shown to be optimal [19, 20], although a single SNP has been suggested to be adequate in European-descent individuals [21]. CRC risk was hypothesized to be elevated in individuals who are rapid acetylators. Multiple studies have reported a stronger association between CRC or adenoma among individuals with the rapid acetylator phenotype or genotype, although this was not observed in all studies. In fact, meta-analyses of the main effect associations of NAT2 acetylator status and colorectal neoplasm have not confirmed this association [22–24].

Additional studies have reported an interaction between intake of red meat or well-done meat, or HAA, and NAT2 acetylator status on the risk of colorectal neoplasia [25–30]. However, other studies failed to replicate this interaction [31–33]. The current status of the evidence for a combined role of HAA and NAT2 in CRC is arguably best captured by two large, carefully conducted, pooled-analyses of individual-level data, one focusing on studies of European descent individuals [34], the other on studies of Japanese and African Americans [35]. In each of the two reports, exposure and genotype data were carefully harmonized and adjustment was carried out for potential confounders.

The first study used the data from the Colon Cancer Family Registry and the Genetics and Epidemiology of Colorectal Cancer Consortium with a total of 8290 CRC cases and 9115 controls from 11 individual studies [34]. NAT2 phenotype was inferred using a single SNP (rs1495741) reported to predict the NAT2 slow phenotype with 99% sensitivity and 95% specificity in whites [21]. Overall, the inferred NAT2 phenotype was not associated with CRC [34]. Red meat intake was collected by each component study using a variety of questionnaires and was found to be associated with risk in a dose-dependent fashion. However, this main effect association was observed only in case-control studies when diet was assessed after diagnosis, not in prospective studies with diet assessed prospectively before diagnosis. Overall, there was no interaction between red meat intake and *NAT2*, as similar associations with CRC were found with red meat for individuals with the combined rapid/intermediate NAT2 phenotype as for those with the slow NAT2 phenotype (Table 1) (p_interaction_ = 0.99). No result was given separately for the rapid phenotype.

**Table 1:** Association (Odds Ratios and 95% Confidence Intervals) between Red Meat Intake and Colorectal Cancer according to Inferred NAT2 phenotype in two pooled analyses

			Red Meat Intake				
Population	NAT2	Case/Controls	Quartile 1	Quartile 2	Quartile 3	Quartile 4	P_trend_
White [34]	S	4906/5488	1.0	1.15 1.03–1.28	1.30 1.17–1.46	1.43 1.28–1.61	NA
	R/I	3384/3627	1.0	1.15 1.11–1.46	1.27 1.11–1.46	1.38 1.20–1.59	NA
			P_interaction_ = 0.97				
Japanese + African Americans [35]	S	295/1057	1.0	1.28 0.84–1.93	1.04 0.69–1.58	0.92 0.60–1.40	0.53
	I	1288/4829	1.0	1.30 1.06–1.59	1.17 0.96–1.43	1.35 1.10–1.65	0.01
	R	1069/2206	1.0	1.21 0.96–1.51	1.16 0.92–1.45	1.47 1.17–1.86	0.003
			P_interaction_ = 0.03				

NAT2 phenotype: S = Slow; I=Intermediate; R = Rapid.

The second study [35] focused on two populations (Japanese and African Americans) with high rates of CRC and with a prevalence of the at-risk, rapid NAT2 phenotype 10- and 2-fold greater than in whites, respectively. Four colorectal cancer studies conducted among Japanese (2217 cases; 3788 controls) and three among African Americans (527 cases; 4527 controls) were meta-analyzed. NAT2 phenotype was inferred from an optimized seven-SNP score [19, 20]. Red meat intake was associated, overall, with risk of CRC (*p* = 0.001), whereas the genetically-inferred NAT2 phenotype was not. A statistically significant interaction was observed between red meat intake and NAT2 activity in both populations combined (p_interaction_ = 0.03), with the association of red meat with CRC being strongest among individuals with the rapid NAT2 phenotype, intermediate among those with the intermediate phenotype and non-significant among those with slow NAT2 phenotype (Table 1). This interaction was suggested in each population, but more strongly in Japanese [35].

Taken at face value, these two studies would suggest that the modifying effect of NAT2 on the association between red meat intake and CRC may be population specific and may only exist in populations with a high prevalence of the rapid acetylation phenotype, such as Japanese and, to a lesser extent, African Americans. This is biologically plausible since NAT2 appears to play a crucial role in the genotoxicity of HAAs. *N*-hydroxylated HAA metabolites are substrates for *O*-acetylation primarily by NAT2 to form reactive *N*-acetoxy species which can bind to DNA [2]. As the result, cancer risk may be particularly elevated in individual who are rapid acetylators. Other population differences that may be at play include cooking and eating practices for meat and fish. Most likely, risk differences result from a combination of both genetics and HAA intake levels. On-going efforts to develop biomarkers of long-term exposure to HAAs may prove crucial in clarifying the role of HAA in human cancer.

### Biomarkers for HAA exposure and intermediate effects

Molecular epidemiology has provided a paradigm to strengthen the evidence for the causal role of a chemical carcinogen in human cancer. The use of biochemical assays to determine the level of exposure and the presence of chemical-specific DNA adducts in target tissues, combined with the correlation of these DNA adducts with specific somatic mutation spectra in tumor related genes, provides a mechanistic blueprint for the causal role of a chemical in the development of cancer [36].

With regard to biomarkers of exposure, the measurement of the HAA 2-amino-1-methyl-6-phenylimidazo [4,5-*b*] pyridine (PhIP) in hair, adjusted for melanin hair content, seems to be the most promising for use in molecular epidemiologic studies. PhIP measured in the hair represents an integrated exposure over a time period of weeks to months and has been shown to be relatively constant over time. Feeding studies with well-done red meat have shown a very good correlation between ingested dose and PhIP hair level [37, 38]. However, this hair biomarker has not been found to be predictor of DNA damage in circulating lymphocytes [39]. The development of highly sensitive quantitative methods to measure DNA adducts [2, 40] and a rapid high-throughput method to extract DNA from formalin-fixed paraffin-embedded tissues [41] which allows measurement of PhIP DNA adducts in widely available archived tissue samples will facilitate the conduct of large-scale studies to measure DNA adduct levels in the target organ in cancer patients.

## Conclusion

Much research have been conducted on the role of HAA in human cancer since Dr. Sugimura’s discovery of HAA in his wife’s broiled fish. Colorectal cancer has been the most studied by epidemiologists. The difficulty in assessing HAA exposure from diet has resulted in inconsistent findings. Focusing on genetically susceptible individuals was favored to demonstrate a link with cancer. Two recent large pooled analyses of colorectal cancer studies, one of European-descent individuals, the other of Japanese and African Americans, have suggested that the modifying effect of NAT2 on the association between red meat intake and CRC may be limited to populations with a high prevalence of the rapid acetylation phenotype (e.g., Japanese and African Americans). In those groups, the association of red meat with colorectal cancer was found to be strongest among individuals with the rapid NAT2 phenotype, intermediate among those with the intermediate phenotype and non-significant among those with slow NAT2 phenotype. Recent research on biomarkers have focused on PhIP hair content to assess usual exposure to HAA and on DNA adducts using new sensitive quantitative methods to demonstrate early biological effects. These studies, when matured, have the potential to contribute greatly to the further elucidation of the carcinogenicity of HAA in humans.

## Data Availability

Not applicable.

## Abbreviations

CRC: Colorectal cancer
CYP: Cytochrome P450
HAA: Heterocyclic aromatic amines
NAT: *N*-acetyl-transferase
PhIP: 2-amino-1-methyl-6-phenylimidazo[4,5-*b*]pyridine

## References

[1] Sugimura T, Wakabayashi K, Nakagama H, Nagao M (2004). Heterocyclic amines: mutagens/carcinogens produced during cooking of meat and fish. Cancer Sci.

[2] Turesky RJ, Le Marchand L (2011). Metabolism and biomarkers of heterocyclic aromatic amines in molecular epidemiology studies: lessons learned from aromatic amines. Cem Res Toxicol.

[3] Bouvard V, Loomis D, Guyton KZ, Grosse Y, Ghissassi FE, Benbrahim-Tallaa L, Guha N, Mattock H, Straif K, International Agency for Research on Cancer Monograph Working Group (2015). International Agency for Research on Cancer monograph working group. Carcinogenicity of consumption of red and processed meat. Lancet Oncol.

[4] Sinha R, Kulldorff M, Chow WH, Denobile J, Rothman N (2001). Dietary intake of heterocyclic amines, meat-derived mutagenic activity, and risk of colorectal adenomas. Cancer Epidemiol Biomark Prev.

[5] Nowell S, Coles B, Sinha R, MacLeod S, Luke Ratnasinghe D, Stotts C (2002). Analysis of total meat intake and exposure to individual heterocyclic amines in a case-control study of colorectal cancer: contribution of metabolic variation to risk. Mutat Res.

[6] Butler LM, Sinha R, Millikan RC, Martin CF, Newman B, Gammon MD, Ammerman AS, Sandler RS (2003). Heterocyclic amines, meat intake, and association with colon cancer in a population-based study. Am J Epidemiol.

[7] Wu K, Giovannucci E, Byrne C, Platz EA, Fuchs C, Willett WC (2006). Meat mutagens and risk of distal colon adenoma in a cohort of U.S. men. Cancer Epidemiol Biomark Prev.

[8] Augustsson K, Skog K, Jägerstad M, Dickman PW, Steineck G (1999). Dietary heterocyclic amines and cancer of the colon, rectum, bladder, and kidney: a population-based study. Lancet..

[9] Ollberding NJ, Wilkens LR, Henderson BE, Kolonel LN, Le Marchand L (2012). Meat consumption, heterocyclic amines and colorectal cancer risk: the multiethnic cohort study. Int J Cancer.

[10] Lang NP, Butler MA, Massengill J, Lawson M, Stotts RC, Hauer-Jensen M (1994). Rapid metabolic phenotypes for acetyltransferase and cytochrome P4501A2 and putative exposure to food-borne heterocyclic amines increase the risk for colorectal cancer or polyps. Cancer Epidemiol Biomark Prev.

[11] Le Marchand L, Hankin JH, Wilkens LR, Pierce LM, Franke A, Kolonel LN (2001). Combined effects of well-done red meat, smoking, and rapid N-acetyltransferase 2 and CYP1A2 phenotypes in increasing colorectal cancer risk. Cancer Epidemiol Biomark Prev.

[12] Le Marchand L, Hankin JH, Pierce LM, Sinha R, Nerurkar PV, Franke AA (2002). Well-done red meat, metabolic phenotypes and colorectal cancer in Hawaii. Mutat Res.

[13] Ishibe N, Sinha R, Hein DW, Kulldorff M, Strickland P, Fretland AJ, Chow WH, Kadlubar FF, Lang NP, Rothman N (2002). Genetic polymorphisms in heterocyclic amine metabolism and risk of colorectal adenomas. Pharmacogenetics.

[14] Ellard GA (1976). Variations between individuals and populations in the acetylation of isoniazid and its significance for the treatment of pulmonary tuberculosis. Clin Pharmacol Ther.

[15] Bray F, Ferlay J, Soerjomataram I, Siegel RL, Torre LA, Jemal A (2018). Global cancer statistics 2018: GLOBOCAN estimates of incidence and mortality worldwide for 36 cancers in 185 countries. CA Cancer J Clin.

[16] Miller BA, Kolonel LN, Bernstein L, Young JJL, Swanson GM, West D (1996). Racial/ethnic patterns of Cancer in the United States 1988–1992.

[17] Kono S (2004). Secular trends of colon cancer incidence and mortality in relation to fat and meat intake in Japan. Eur J Cancer Prev.

[18] Ognjanovic S, Yamamoto J, Maskarinec G, Le Marchand L (2006). NAT2, meat consumption and colorectal cancer incidence: an ecological study among 27 countries. Cancer Causes Control.

[19] Doll MA, Hein DW (2001). Comprehensive human NAT2 genotype method using single nucleotide polymorphism-specific polymerase chain reaction primers and fluorogenic probes. Anal Biochem.

[20] Deitz AC, Rothman N, Rebbeck TR, Hayes RB, Chow WH, Zheng W (2004). Impact of misclassification in genotype-exposure interaction studies: example of N-acetyltransferase 2 (NAT2), smoking, and bladder cancer. Cancer Epidemiol Biomark Prev.

[21] García-Closas M, Hein DW, Silverman D, Malats N, Yeager M, Jacobs K, Doll MA, Figueroa JD, Baris D, Schwenn M, Kogevinas M, Johnson A, Chatterjee N, Moore LE, Moeller T, Real FX, Chanock S, Rothman N (2011). A single nucleotide polymorphism tags variation in the arylamine N-acetyltransferase 2 phenotype in populations of European background. Pharmacogenet Genomics.

[22] Brockton N, Little J, Sharp L, Cotton SC (2000). N-acetyltransferase polymorphisms and colorectal cancer: a HuGE review. Am J Epidemiol.

[23] Liu J, Ding D, Wang X, Chen Y, Li R, Zhang Y, Luo R (2012). N-acetyltransferase polymorphism and risk of colorectal adenoma and cancer: a pooled analysis of variations from 59 studies. PLoS One.

[24] Zhang L, Zhou J, Wang J, Liang G, Li J, Zhu Y (2012). Absence of association between N-acetyltransferase 2 acetylator status and colorectal cancer susceptibility: based on evidence from 40 studies. PLoS One.

[25] Welfare MR, Cooper J, Bassendine MF, Daly AK (1997). Relationship between acetylator status, smoking, and diet and colorectal cancer risk in the north-east of England. Carcinogenesis.

[26] Kampman E, Slattery ML, Bigler J, Leppert M, Samowitz W, Caan BJ (1999). Meat consumption, genetic susceptibility, and colon cancer risk: a United States multicenter case-control study. Cancer Epidemiol Biomark Prev.

[27] Chan AT, Tranah GJ, Giovannucci EL, Willett WC, Hunter DJ, Fuchs CS (2005). Prospective study of N-acetyltransferase-2 genotypes, meat intake, smoking and risk of colorectal cancer. Int J Cancer.

[28] Lilla C, Verla-Tebit E, Risch A, Jäger B, Hoffmeister M, Brenner H (2006). Effect of NAT1 and NAT2 genetic polymorphisms on colorectal cancer risk associated with exposure to tobacco smoke and meat consumption. Cancer Epidemiol Biomark Prev.

[29] Nöthlings U, Yamamoto JF, Wilkens LR, Murphy SP, Park SY, Henderson BE, Kolonel LN, le Marchand L (2009). Meat and heterocyclic amine intake, smoking, NAT1 and NAT2 polymorphisms, and colorectal cancer risk in the multiethnic cohort study. Cancer Epidemiol Biomark Prev.

[30] Voutsinas J, Wilkens LR, Franke A, Vogt TM, Yokochi LA, Decker R, le Marchand L (2013). Heterocyclic amine intake, smoking, cytochrome P450 1A2 and N-acetylation phenotypes, and risk of colorectal adenoma in a multiethnic population. Gut..

[31] Murtaugh MA, Ma KN, Sweeney C, Caan BJ, Slattery ML (2004). Meat consumption patterns and preparation, genetic variants of metabolic enzymes, and their association with rectal cancer in men and women. J Nutr.

[32] Budhathoki S, Iwasaki M, Yamaji T, Sasazuki S, Takachi R, Sakamoto H, Yoshida T, Tsugane S (2015). Dietary heterocyclic amine intake, NAT2 genetic polymorphism, and colorectal adenoma risk: the colorectal adenoma study in Tokyo. Cancer Epidemiol Biomark Prev.

[33] Barrett JH, Smith G, Waxman R, Gooderham N, Lightfoot T, Garner RC, Augustsson K, Wolf CR, Bishop DT, Forman D, Colorectal Cancer Study Group (2003). Colorectal Cancer study group. Investigation of interaction between N-acetyltransferase 2 and heterocyclic amines as potential risk factors for colorectal cancer. Carcinogenesis.

[34] Ananthakrishnan AN, Du M, Berndt SI, Brenner H, Caan BJ, Casey G (2015). Red meat intake, NAT2, and risk of colorectal cancer: a pooled analysis of 11 studies. Cancer Epidemiol Biomark Prev.

[35] Wang H, Iwasaki M, Haiman CA, Kono S, Wilkens LR, Keku TO (2015). Interaction between red meat intake and NAT2 genotype in increasing the risk of colorectal Cancer in Japanese and African Americans. PLoS One.

[36] Jarabek AM, Pottenger LH, Andrews LS, Casciano D, Embry MR, Kim JH, Preston RJ, Reddy MV, Schoeny R, Shuker D, Skare J, Swenberg J, Williams GM, Zeiger E (2009). Creating context for the use of DNA adduct data in cancer risk assessment: I. Data organization Crit Rev Toxicol.

[37] Turesky RJ, Liu L, Gu D, Yonemori KM, White KK, Wilkens LR, le Marchand L (2013). Biomonitoring the cooked meat carcinogen 2-amino-1-methyl-6-phenylimidazo[4,5-b] pyridine in hair: impact of exposure, hair pigmentation, and cytochrome P450 1A2 phenotype. Cancer Epidemiol Biomark Prev.

[38] Le Marchand L, Yonemori K, White KK, Franke AA, Wilkens LR, Turesky RJ (2016). Dose validation of PhIP hair level as a biomarker of heterocyclic aromatic amines exposure: a feeding study. Carcinogenesis.

[39] Bessette EE, Yasa I, Dunbar D, Wilkens LR, Le Marchand L, Turesky RJ (2009). Biomonitoring of carcinogenic heterocyclic aromatic amines in hair: a validation study. Chem Res Toxicol.

[40] Balbo S, Turesky RJ, Villalta PW (2014). DNA adductomics. Chem Res Toxicol.

[41] Yun BH, Xiao S, Yao L, Krishnamachari S, Rosenquist TA, Dickman KG (2017). A Rapid Throughput Method To Extract DNA from Formalin-Fixed Paraffin-Embedded Tissues for Biomonitoring Carcinogenic DNA Adducts. Chem Res Toxicol.

